# Prospective, randomized, single-blinded, multi-center phase II trial of two HER2 peptide vaccines, GP2 and AE37, in breast cancer patients to prevent recurrence

**DOI:** 10.1007/s10549-020-05638-x

**Published:** 2020-04-22

**Authors:** Tommy A. Brown, Elizabeth A. Mittendorf, Diane F. Hale, John W. Myers, Kaitlin M. Peace, Doreen O. Jackson, Julia M. Greene, Timothy J. Vreeland, G. Travis Clifton, Alexandros Ardavanis, Jennifer K. Litton, Nathan M. Shumway, J. Symanowski, James L. Murray, Sathibalan Ponniah, E. A. Anastasopoulou, N. F. Pistamaltzian, Constantin N. Baxevanis, Sonia A. Perez, Michael Papamichail, George E. Peoples

**Affiliations:** 1 Department of Surgery, Brooke Army Medical Center, Ft. Sam Houston, San Antonio, TX USA; 2 Department of Breast Surgical Oncology, The University of Texas MD Anderson Cancer Center, Houston, TX USA; 3 Department of Surgical Oncology, The University of Texas MD Anderson Cancer Center, Houston, TX USA; 4 Cancer Immunology and Immunotherapy Center, St. Savas Cancer Hospital, Athens, Greece; 5 Department of Breast Medical Oncology, The University of Texas MD Anderson Cancer Center, Houston, TX USA; 6 Texas Oncology PA, San Antonio, TX USA; 7 Department of Cancer Biostatistics, Levine Cancer Institute, Charlotte, NC USA; 8 Cancer Vaccine Development Laboratory, Department of Surgery, Uniformed Services University of the Health Sciences, Bethesda, MD USA; 9 Department of Surgery, Uniformed Services Health University, Bethesda, MD USA; 10 Present Address: Division of Breast Surgery, Department of Surgery, Breast Oncology Program, Brigham and Women’s Hospital, Dana-Farber/Brigham and Women’s Hospital, Boston, MA USA; 11 Cancer Vaccine Development Program, 1305 East Houston Street, San Antonio, TX 78205 USA

**Keywords:** Immunotherapy, Vaccine, Breast cancer, HER2, AE37, GP2

## Abstract

**Purpose:**

AE37 and GP2 are HER2 derived peptide vaccines. AE37 primarily elicits a CD4+ response while GP2 elicits a CD8+ response against the HER2 antigen. These peptides were tested in a large randomized trial to assess their ability to prevent recurrence in HER2 expressing breast cancer patients. The primary analyses found no difference in 5-year overall disease-free survival (DFS) but possible benefit in subgroups. Here, we present the final landmark analysis.

**Methods:**

In this 4-arm, prospective, randomized, single-blinded, multi-center phase II trial, disease-free node positive and high-risk node negative breast cancer patients enrolled after standard of care therapy. Six monthly inoculations of vaccine (VG) vs. control (CG) were given as the primary vaccine series with 4 boosters at 6-month intervals. Demographic, safety, immunologic, and DFS data were evaluated.

**Results:**

456 patients were enrolled; 154 patients in the VG and 147 in CG for AE37, 89 patients in the VG and 91 in CG for GP2. The AE37 arm had no difference in DFS as compared to CG, but pre-specified exploratory subgroup analyses showed a trend towards benefit in advanced stage (*p* = 0.132, HR 0.573 CI 0.275–1.193), HER2 under-expression (*p* = 0.181, HR 0.756 CI 0.499–1.145), and triple-negative breast cancer (*p* = 0.266, HR 0.443 CI 0.114–1.717). In patients with both HER2 under-expression and advanced stage, there was significant benefit in the VG (*p* = 0.039, HR 0.375 CI 0.142–0.988) as compared to CG. The GP2 arm had no significant difference in DFS as compared to CG, but on subgroup analysis, HER2 positive patients had no recurrences with a trend toward improved DFS (*p* = 0.052) in VG as compared to CG.

**Conclusions:**

This phase II trial reveals that AE37 and GP2 are safe and possibly associated with improved clinical outcomes of DFS in certain subgroups of breast cancer patients. With these findings, further evaluations are warranted of AE37 and GP2 vaccines given in combination and/or separately for specific subsets of breast cancer patients based on their disease biology.

## Introduction

Despite progress via early detection and improved treatment, breast cancer recurrence remains a significant problem. Immunotherapy shows promise in the treatment of multiple cancers and may further improve outcomes in breast cancer. Increasing evidence suggests breast cancer is more immunogenic than once realized, particularly given the important prognostic role that the host immune response and tumor microenvironment play [1–3]. Immune-mediated surveillance and clearance of disease likely plays an important role in preventing recurrence in clinically disease-free patients after standard of care therapy. Cancer vaccines may help generate a tumor-specific immunity to prevent disease recurrence.

HER2 is a tumor-associated antigen expressed at some level in 60–70% of breast cancers, over-expressed in 20–30% of patients, and is one potential target for breast cancer vaccines [4, 5]. Monoclonal antibodies targeting HER2 provide clinical benefit, at least in part due to an immunologic mechanism in HER2 over-expressing breast cancer [6]. Likewise, vaccines targeting immunogenic HER2 peptides may provide benefit via immune-mediated cancer cell elimination.

The HER2-specific vaccine nelipepimut-S (HER2 369–377, E75, NeuVax) is a human leukocyte antigen (HLA) A2 restricted, major histocompatibility complex (MHC) class I, dominant epitope derived from the extracellular domain of HER2, and has been evaluated in the adjuvant setting to prevent breast cancer recurrence in women rendered clinically disease-free after standard-of-care therapy [7–9]. Nelipepimut-S induces a CD8+ cytotoxic T-lymphocyte (CTL) response to HER2. In phase II trials, nelipepimut-S was found to be safe, effective in raising HER2-specific immunity, and showed evidence of improved disease-free survival [8] However, a phase III trial of nelipepimut-S in the adjuvant setting was stopped early for futility [10].

A single, dominant CD8+ CTL targeted epitope may not be an effective strategy for all breast cancer patients. Just as distinct biologic subtypes of breast cancer are better served by different conventional therapies, they may also benefit from unique vaccine strategies. Thus, exploring additional strategies, such as, MHC class-II epitope treatment stimulating a CD4+ T helper cell response [11–13] and treatment with subdominant epitopes, which may be less prone to T-cell anergy by persistent antigen exposure may be beneficial [14].

One of our efforts to explore additional vaccination strategies with broader applicability is the AE37 peptide vaccine. AE37 is an MHC class-II peptide that is a modified version of the naturally occurring AE36 wild-type peptide (HER2 776–790) derived from the intracellular domain of HER2 with the addition of the 4 amino acid long Ii-Key peptide (LRMK). The Ii-key peptide is added to enhance immunogenicity to AE36 [15]. Additionally, AE37 is HLA unrestricted, allowing it to be used in a broader population of patients.

In another vaccination strategy effort to avoid over-stimulation and anergy, we have tested a subdominant immunogenic peptide, GP2 (HER2 654–662). GP2 is an HLA-A2 restricted immunogenic peptide derived from the transmembrane domain of HER2. While GP2 has a lower affinity to HLA-A2 than nelipepimut-S, it has been shown to be as efficacious as nelipepimut-S in inducing a CTL response [16].

We have previously published primary analyses from our large randomized trial of the AE37 and GP2 peptides. There was no demonstrable difference in 5-year overall disease-free survival (DFS) in the intention-to-treat populations. However, there was evidence of benefit in subgroups of breast cancer patients [7, 17, 18]. Here, we present the final analysis of the primary endpoint of DFS with additional follow-up as well as additional per-treatment analysis, along with comprehensive pre-specified subset analyses for both the GP2 and AE37 peptide vaccines used in a randomized controlled trial of breast cancer patients with any level of HER2 expression that were clinically disease-free and at a high risk for recurrence.

## Methods

### Patient characteristics and clinical protocol

The study was designed as a 4-arm, prospective, randomized, single-blinded, multi-center phase II trial (NCT00524277), conducted under the investigational new drug applications BB-IND #12229 and #11730. Clinically disease-free node positive and high-risk node negative breast cancer patients were enrolled one to six months after completion of primary standard of care therapy with the exception of adjuvant endocrine therapy which was allowed concurrently. High-risk node negative patients were defined if they had any of the following: as ≥ T2, grade 3, lymphovascular invasion, estrogen or progesterone receptor negative, HER2 over-expressing tumor (IHC 3+ and/or amplified FISH > 2.0, before CAP/ASCO guideline changes), or N0 (i+) breast cancer patients with any level of HER2 expression (IHC 1–3+ and/or positive FISH > 1.2). HLA-A2 positive patients were assigned to either the GP2 or AE37 arms of the trial, both given in combination with granulocyte–macrophage colony-stimulating factor (GM-CSF) in the treatment groups or GM-CSF alone in the control group. HLA-A2 negative patients were randomized to receive either AE37 in combination with GM-CSF in the treatment group or GM-CSF alone in the control group.

The primary objectives of this study were to determine if AE37 in combination with GM-CSF vaccination improves the DFS in any level HER2 expressing, node positive or high-risk node negative breast cancer patients, and to determine if GP2 in combination with GM-CSF vaccination improves the DFS in any level HER2 expressing, HLA-A2 positive, node positive or high-risk node negative breast cancer patients. In addition, the DFS were compared between all four arms of the trial. Based on our previous trials with nelipepimut-S, the difference in recurrence was 15% in controls compared with 6% in the vaccinated patients at a median follow-up of 2 years [9]. Based on this data, the trial was designed to detect a 0.48 hazard ratio corresponding to an improvement in 2-year DFS from 85% for GM-CSF control to 93% for vaccine (AE37 and GP2). A sample size of 150 subjects per group had 80% power to detect the difference at a 1-sided alpha level of 0.05 using a log-rank test for equality of survival curves. The total number of events required to achieve the specified power was 33. A sample size of 100 subjects per group would have the same power to detect a statistical difference between groups with a hazard ratio of 0.35.

Twenty-five HLA-A2 positive patients who were assigned to the AE37 arm of the trial and randomized to the control group were included in the analysis of both the AE37 and GP2 arms as controls. Their inclusion is justified based on an evaluation of clinical outcomes in the control patients confirming that HLA-A2-status does not affect DFS regardless of HER2 expression [19].

### Vaccine and vaccination series

The GP2 and AE37 peptides were created in keeping with good manufacturing practices and purified to > 95%. Sterility, endotoxin and general safety testing were performed prior to administration. Six inoculations were given in 3–4 week intervals administered intradermally consistently in the same lymph node distribution (same arm or thigh) in each patient. Patients in each treatment arm received 500 mcg of the peptide and 125 mcg of GM-CSF, while the control arm received 125 mcg of GM-CSF alone. After the initial 6 inoculations, patients were given a total of 4 booster inoculations at six-month intervals beginning one year after each subject’s date of enrollment (at 12, 18, 24, and 30 months).

### Clinical recurrence of disease

The patients’ primary physicians determined recurrence, the primary endpoint, at their individual study sites during routine follow-up. All enrolled patients were evaluated every 3 months for the first 24 months after completion of primary therapies, and every 6 months for an additional 36 months with clinical exam, laboratory, and radiographic surveillance as per standard of care. All enrolled patients were followed for clinical recurrence for up to 5 years; one site offered extended voluntary follow-up beyond 5 years for the patients randomized into the AE37 arm of the trial.

### Statistical analysis

Patients were stratified by site and by nodal status then randomized into treatment groups in a 1:1 allocation ratio. Clinicopathologic data were compared between groups with median and interquartile range used to summarize age. The groups were then compared using analysis of variance techniques. Categorical variables were summarized with frequencies and proportions. Groups were compared using a two-sided Fisher’s exact test and Forest plot. DFS was calculated from randomization date to recurrence date or death due to any cause. Data were censored by the date of last contact. DFS was analyzed using the Kaplan–Meier method with log-rank comparisons. Cox proportional hazard models were used to estimate hazard ratios and 95% confidence intervals to estimate the relative risk of recurrence or death between arms. Per-treatment (PT) analyses excluded patients with early recurrences (before completion of the primary vaccine series) and those who developed second malignancies.

## Results

### Demographics

**Fig. 1:**
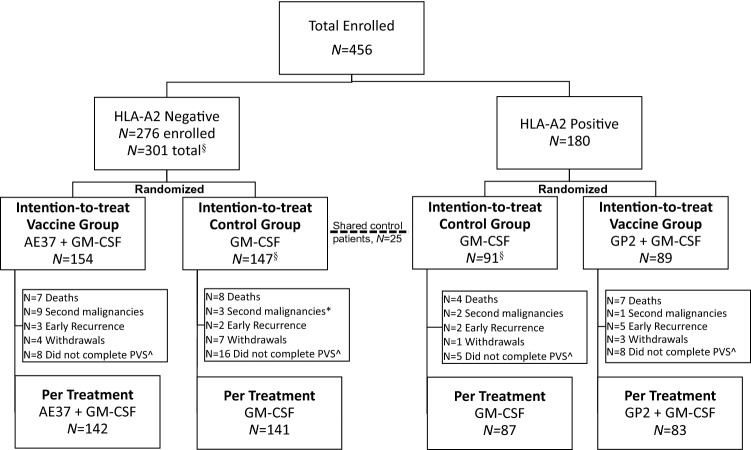
Consort diagram. ^§^25 HLA-A2 positive patients were used as controls in both groups. *1 patient with a second malignancy in the AE37 control arm withdrew and is also included in the 7 withdrawals. ^^^Defined as patients that did not complete the PVS, patients that withdrew, met the primary end point (recurrence, second malignancy, or death from any cause), or chose not to continue on study before completing the PVS

A total of 456 patients were enrolled at 16 sites throughout the United States between 2007 and 2013. Both HLA-A2 positive and negative patients were eligible for enrollment in the AE37 arm. For a portion of the enrollment period, all patients were enrolled in the AE37 arm, while during the remainder, HLA-A2 negative patients were enrolled in the AE37 arm and HLA-A2 positive patients were enrolled in the GP2 arm. In the AE37 arm, patients were randomized to receive either AE37 in combination with GM-CSF (*n* = 154 total; HLA-A2 positive *n* = 24, HLA-A2 negative *n* = 130) or GM-CSF alone (*n* = 147 total, HLA-A2 positive *n* = 25, HLA-A2 negative *n* = 122). A total of 180 HLA-A2 positive patients were randomized to receive either GP2 in combination with GM-CSF (*n* = 89) or GM-CSF alone (*n* = 91). Twenty-five HLA-A2 positive GM-CSF only control patients initially enrolled in the AE37 arm were included as control patients in the GP2 arm (Fig. 1). The per-treatment analysis excluded patients with second malignancy and early recurrence. Within the GP2 PT analysis there were 10 patients excluded; 6 from the vaccine group and 4 from the control. Within the AE37 PT analysis there were 17 patients excluded; 12 from the vaccine group and 5 from the control. There were no significant clinical or pathologic differences between the treatment and controls groups for either the GP2 (Table 1) or AE37 (Table 2) arms.

**Table 1:** GP2 demographics

	Vaccine (*n* = 89) *n* (%)	Control *(n* = 91) *n* (%)	*p* value
Median age (years)	50.8	51.1	0.928
IQR	44.3–57.6	44.4–58.6	
T stage			0.702
T0	2 (2.2)	2 (2.2)	
Tis/mic	1 (1.1)	2 (2.2)	
T1	40 (44.9)	39 (42.9)	
T2	34 (38.2)	33 (36.3)	
T3	6 (6.7)	11 (12.1)	
T4	4 (4.5)	4 (4.4)	
Tx	2 (2.2)	0 (0)	
Nodal status			0.234
Positive	51 (57.3)	60 (65.9)	
Negative	38 (42.7)	31 (34.1)	
Histology			0.094
Ductal	84 (94.4)	77 (84.6)	
Lobular	2 (2.2)	4 (4.4)	
Other	3 (3.4)	10 (11.0)	
Grade (differentiation)			0.339
Well	4 (4.5)	9 (9.9)	
Moderate	34 (38.2)	30 (33.0)	
Poor	51 (57.3)	52 (57.1)	
ER/PR status			0.564
Positive	55 (61.8)	60 (65.9)	
Negative	34 (38.2)	31 (34.1)	
HER2 status			0.750
Positive	51 (57.3)	50 (54.9)	
Negative	38 (42.7)	41 (45.1)	
Surgery			0.341
Lumpectomy	36 (40.4)	33 (36.3)	
Mastectomy	46 (51.7)	54 (59.3)	
Both	7 (7.9)	3 (3.3)	
None	0 (0)	1 (1.1)	
Radiation therapy			0.418
Adjuvant	67 (75.3)	74 (81.3)	
Neoadjuvant	1 (1.1)	0 (0)	
None	21 (23.6)	17 (18.7)	
Chemotherapy			0.668
Adjuvant	65 (73.0)	69 (75.8)	
Neoadjuvant	16 (18.0)	14 (15.4)	
Adjuvant and neoadjuvant	1 (1.1)	3 (3.3)	
None	7 (7.9)	5 (5.5)	
Trastuzumab therapy			0.808
Adjuvant	40 (44.9)	38 (41.8)	
Neoadjuvant	4 (4.5)	3 (3.3)	
Adjuvant and neoadjuvant	3 (3.4)	4 (4.4)	
None	42 (47.2)	46 (50.5)	
Endocrine therapy			0.482
Aromatase inhibitor	29 (32.6)	29 (31.9)	
Tamoxifen	24 (27.0)	29 (31.9)	
Other	2 (2.2)	0 (0)	
None	34 (38.2)	33 (36.3)

**Table 2:** AE37 demographics

	Vaccine (*n* = 154) *n* (%)	Control (*n* = 147) *n* (%)	*p* value
Median age (years)	49.0	50.4	0.503
IQR (years)	42.5–57.6	42.7–57.6	
T stage			0.706
T0	4 (2.6)	3 (2.0)	
Tis/mic	2 (1.3)	3 (2.0)	
T1	64 (41.6)	56 (38.1)	
T2	57 (37.0)	67 (45.6)	
T3	19 (12.3)	14 (9.5)	
T4	5 (3.2)	3 (2.0)	
Tx	3 (1.9)	1 (0.7)	
Nodal status			0.946
Positive	100 (64.9)	96 (65.3)	
Negative	54 (35.1)	51 (34.7)	
Histology			0.486
Ductal	135 (87.7)	124 (84.4)	
Lobular	10 (6.5)	9 (6.1)	
Other	9 (5.8)	14 (9.5)	
Grade (differentiation)			0.788
Well	10 (6.5)	8 (5.4)	
Moderate	66 (42.9)	59 (40.1)	
Poor	78 (50.6)	80 (54.4)	
ER/PR status			0.969
Positive	95 (61.7)	91 (61.9)	
Negative	59 (38.3)	56 (38.1)	
HER2 status			0.443
Positive	77 (50.0)	67 (45.6)	
Negative	77 (50.0)	80 (54.4)	
HLA-A2 status			0.579
Positive	25 (16.2)	25 (17.0)	
Negative	129 (83.8)	121 (82.3)	
Surgery			0.370
Lumpectomy	63 (40.9)	48 (32.7)	
Mastectomy	84 (54.5)	91 (61.9)	
Both	7 (4.5)	7 (4.8)	
None	0 (0)	1 (0.7)	
Radiation therapy			0.775
Adjuvant	118 (76.6)	110 (74.8)	
Neoadjuvant	2 (1.3)	1 (0.7)	
None	34 (22.1)	36 (24.5)	
Chemotherapy			0.403
Adjuvant	109 (70.8)	97 (66.0)	
Neoadjuvant	38 (24.7)	36 (24.5)	
Adjuvant and neoadjuvant	2 (1.3)	4 (2.7)	
None	5 (3.2)	10 (6.8)	
Trastuzumab therapy			0.704
Adjuvant	65 (42.2)	55 (37.4)	
Neoadjuvant	5 (3.2)	4 (2.7)	
Adjuvant and neoadjuvant	6 (3.9)	4 (2.7)	
None	78 (50.6)	84 (57.1)	
Endocrine therapy			0.546
Aromatase inhibitor	46 (29.9)	43 (29.3)	
Tamoxifen	50 (32.5)	47 (32.0)	
Ovarian ablation	1 (0.6)	0 (0)	
Other	2 (1.3)	0 (0)	
None	55 (35.7)	57 (38.8)	

### Safety

**Fig. 2:**
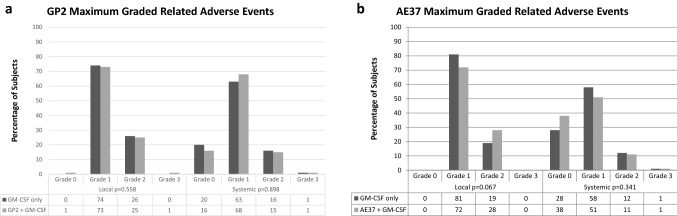
Depicts the maximum graded and related adverse events for the **a** GP2 and **b** AE37 trials

The vaccines were well tolerated with no differences between maximal local (GP2 *p* = 0.558, AE37 *p* = 0.067) or systemic (GP2 *p* = 0.898, AE37 *p* = 0.341) toxicities in either the GP2 (Fig. 2a) or AE37 (Fig. 2b) arms as compared to the controls, this is unchanged from previous reports [7, 17]. A majority of the adverse events were grade 1 in nature; there were no related toxicities greater than grade 3. The similar toxicity profiles between the treatment and control groups across both the GP2 and AE37 arms indicate that the majority of the toxicity can be attributed to the immunoadjuvant, GM-CSF.

### Disease-free survival

**Fig. 3:**
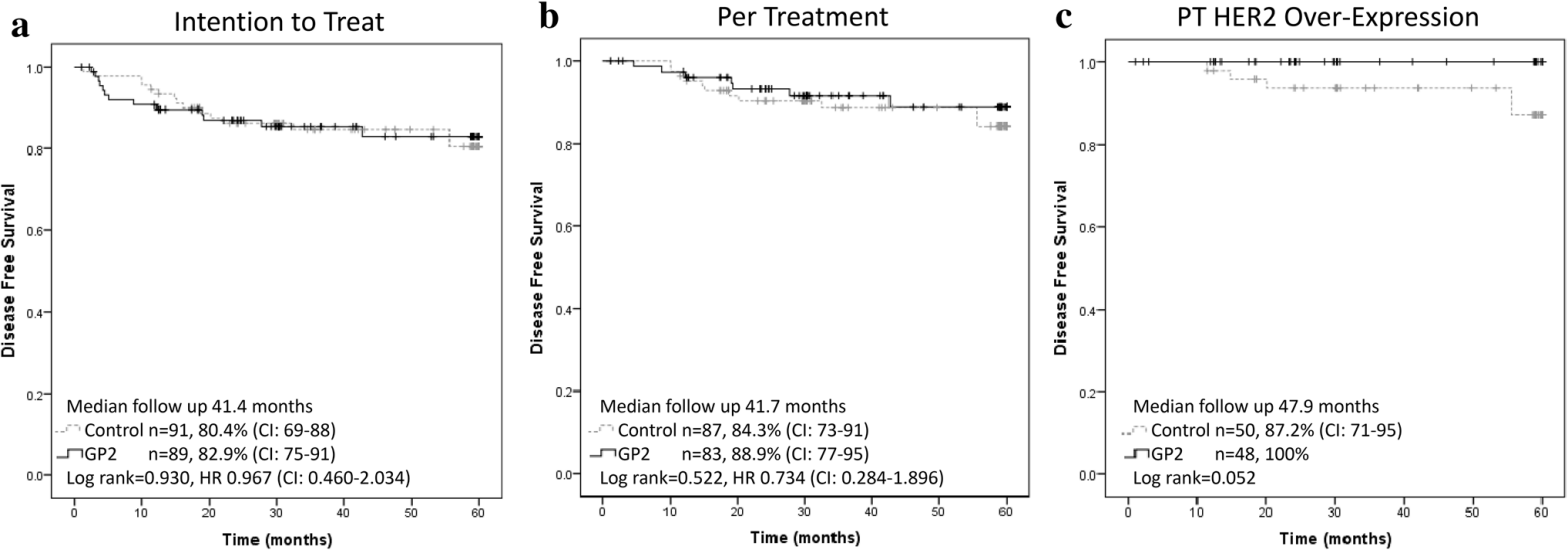
GP2 final 5-year estimated disease-free survival **a** Intention to Treat **b** Per-treatment **c** Per-treatment subset of HER2 Over-expressing breast cancers

**Fig. 4:**
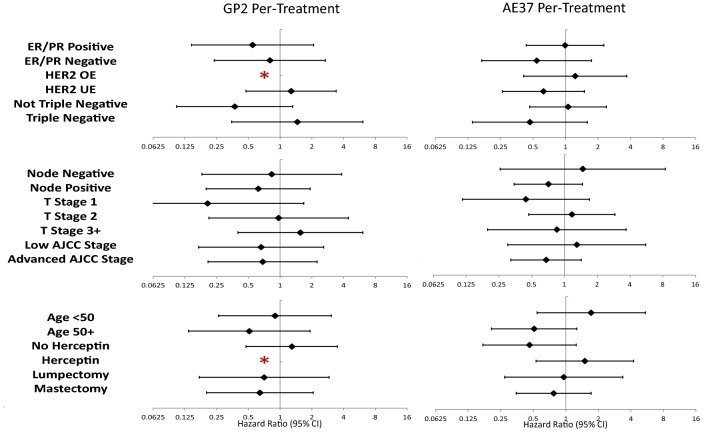
Pre-specified, per-treatment subgroup analysis of histologic (top), pathologic (middle), and patient/treatment (bottom) characteristics looking at relative risk using the Forest plot method with GP2 on the left and AE37 on the right. Advanced AJCC Stage is defined as Stage IIB or greater. *No recurrences in the vaccine group

At the time of the final analysis of the GP2 portion of the trial, the median follow-up was 41.4 (interquartile range [IQR] 24.8–59.2) months for the intention to treat (ITT) and 41.7 (IQR 28.4–59.2) months for the per-treatment (PT), this was approximately 6 months longer than the primary analysis [17]. Similar to the primary analysis, there was no significant difference in 5-year estimated DFS between the vaccine and control arms in either the ITT (82.9% vs 80.4%, *p* = 0.930; hazard ratio [HR] 0.967 95% confidence interval [CI] 0.460–2.034, Fig. 3a) or PT (88.9% vs 84.3%, *p* = 0.522; HR 0.734 CI 0.284–1.896, Fig. 3b) analyses. Upon pre-specified exploratory subgroup analyses of histopathologic, patient, and treatment related characteristics, the HER2 over-expressing patients appeared to derive the greatest benefit from vaccination as there were no recurrences (Fig. 4). In addition, there was a trend toward significant improvement in 5-year estimated DFS among HER2 over-expressing patients receiving GP2 vaccine versus control (100% vs 87.2%, *p* = 0.052, Fig. 3c).

**Fig. 5:**
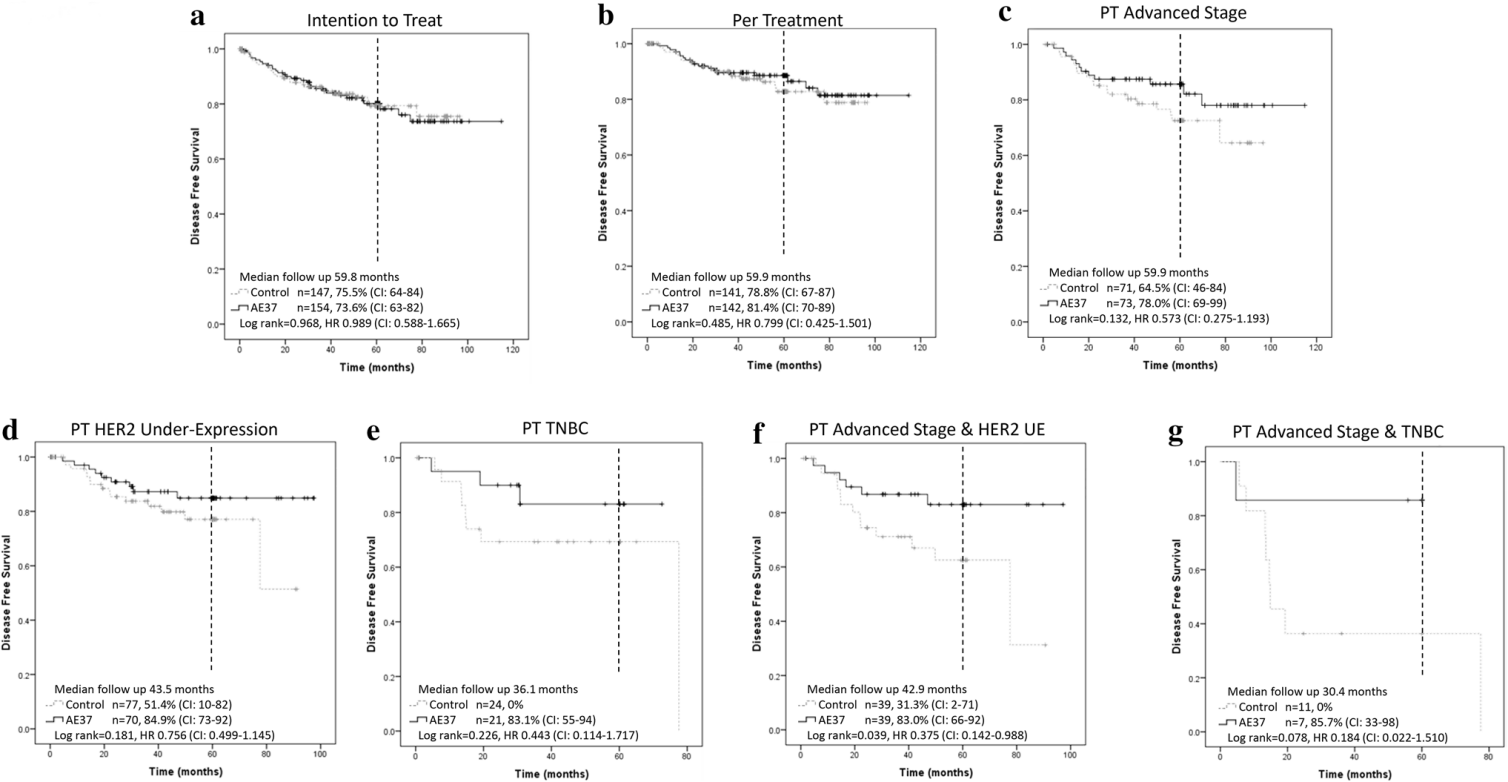
AE37 final 5 to 10-year estimated disease-free survival with vertical dashed line annotating 5-year estimated disease-free survival **a** Intention to Treat **b** Per-treatment **c** Per-treatment subset of advanced stage breast cancers (defined as Stage IIB or greater) **d** Per-treatment subset of HER2 under-expressing cancers **e** Per-treatment subset of triple-negative breast cancers **f** Per-treatment subset of patients with both advanced stage and HER2 under-expressing (defined as HER2 expression IHC 1–2+ and/or positive FISH 1.2–2.0) breast cancers **g** Per-treatment subset of patients with both advanced stage and triple negative breast cancers

In the final analysis of the AE37 portion of the trial, the median follow-up was 59.8 (IQR 37.5–61.7) months for the ITT and 59.9 (IQR 37.9–63.4) months for the PT groups, this was approximately 30 months longer than the primary analysis [7]. Similar to the primary analysis, there was no significant difference in 5-year estimated DFS between the vaccine and control arms in the ITT (80.1% vs 79.3%, *p* = 0.968, HR 0.989 CI 0.588–1.665, Fig. 5a) or PT (88.6% vs 82.8%, *p* = 0.485, HR 0.799 CI 0.425–1.501, Fig. 5b) analyses. Pre-specified exploratory subgroups analyses by histopathologic, patient, and treatment related characteristics showed a trend towards benefit in patients with advanced stage (defined as stage IIB or greater) and HER2 under-expression (HER2 UE, defined as HER2 expression IHC 1–2 + and/or positive FISH 1.2–2.0), and triple-negative breast cancer (TNBC, Fig. 4). This trend was likewise present on 5-year estimated DFS within the advanced stage (AE37 85.7% vs Control 72.5%, *p* = 0.132, HR 0.573 CI 0.275–1.193, Fig. 5c), HER2 under-expression (AE37 84.9% vs Control 77.1%, *p* = 0.181, HR 0.756 CI 0.499–1.145, Fig. 5d), and TNBC (AE37 83.1% vs Control 69.3%, *p* = 0.226, HR 0.443 CI 0.114–1.717, Fig. 5e). In a post hoc analysis of patients with both advanced stage and HER2 under-expression there was a significant improvement in DFS favoring the vaccine group (AE37 83.0% vs Control 62.5%, *p* = 0.039, HR 0.375 CI 0.142–0.988, Fig. 5f). There was a similar trend towards clinical benefit in patients with both advanced stage disease and TNBC (AE37 85.7% vs Control 36.4%, *p* = 0.078, HR 0.184 CI 0.022–1.510, Fig. 5g).

## Discussion

Here, we report the results of a multi-center, randomized, blinded, controlled phase II trial of the peptide vaccines, GP2 and AE37, as adjuvant therapy in women with high-risk breast cancer to prevent recurrence. We found that both the CD8+ CTL-eliciting GP2 and the CD4 + T helper cell-eliciting AE37 vaccines are safe with limited toxicity that is primarily due to the GM-CSF immunoadjuvant and not the individual peptides [7, 17]. Furthermore, while the overall DFS does not appear to be improved in the ITT population, multiple subsets may derive some benefit based on pre-specified exploratory analyses. Interestingly, the responding patient subsets to GP2 and AE37 are very different suggesting the potential to target specific patients and/or combining the peptides to address a broader patient population. A current limitation of this analysis is the per-treatment nature possibly affecting the external validity of the data; although, the number of patients excluded from the ITT to PT analysis was small (*n* = 27, 5.9% overall of patients). Even with the exclusion of these early recurrence and second malignancy patients, there was still no significant differences between the group demographics.

It has long been known that subtypes of breast cancer have different levels of responsiveness to chemotherapy, hormonal therapy, and HER2-directed therapy. Breast cancer subtypes similarly have distinct immunologic characteristics. The TNBC subtype appears to have the greatest amount of immune infiltration, followed by the highly-proliferative estrogen receptor positive subtype. Meanwhile, the low-grade, estrogen receptor positive, luminal A subtype appears to have the lowest infiltration rate [20]. This recognition is of increasing clinical importance, not only because of the recent rise of immunotherapy to the forefront of cancer care, but also because tumor immune characteristics can also be prognostic in breast cancer [21].

The data from this analysis suggest that the GP2 peptide vaccine may be beneficial in patients with HER2 over-expressing tumors who received trastuzumab as part of their standard of care treatment. This supports the hypothesis that GP2 may have synergistic clinical efficacy when combined with trastuzumab [22]. Previous preclinical work by Mittendorf et al. demonstrated that HER2 receptors on the tumor cell surface can be saturated by treatment with trastuzumab, promoting internalization in a time and dose-dependent manner. Trastuzumab increased the sensitivity of the tumor cells to CTL-mediated lysis after stimulation with either nelipepimut-S or GP2, even in patients with low levels of HER2 expression. Interestingly, they also found peripheral blood lymphocytes lyse trastuzumab-treated breast cancer cells more efficiently after nelipepimut-S vaccination. We are currently investigating the possibility of a synergistic immunologic effect when nelipepimut-S is given in combination with trastuzumab in a phase II trial in HER2 over-expressing (3+ by IHC; NCT02297698). We recently completed a trial in low-expressing HER2 (IHC 1–2+) patients and found the greatest clinical benefit in DFS in patients with TNBC, suggesting a synergistic mechanism in this population [23]. In addition to the potential synergy with trastuzumab, GP2 may be inherently more effective in the HER2 over-expressing population. These patients have increased HER2 expression, their immune system has greater exposure to this tumor-associated antigen. Given that GP2 is a subdominant epitope of HER2, there may be less immune tolerance to this epitope than a dominant epitope, such as nelipepimut-S. This likely allows GP2 to be more effective in HER2 over-expressing disease; where nelipepimut-S may be more effective in HER2 low-expressing patients [9].

In the AE37 arm of this trial, we found patients with advanced stage, HER2 under-expression, and TNBC may benefit from AE37 vaccination, and those with both advanced stage and HER2 under-expression have a significant clinical benefit to AE37 vaccination. Specifically, demonstrating earlier DFS plateau that was maintained for up to the ten years of follow-up. AE37 has been shown to induce CD4+ T helper cell stimulation which is required for the effective generation of long-term cell-mediated immunity [24, 25]. Given that the primary response to AE37 is not a CTL response, but instead a CD4+ T helper cell response, the AE37 vaccine may have more of an immunoadjuvant effect to a pre-existing immune response within the patient.

AE37 can also augment a vaccine-induced CTL response. Gates et. al, demonstrated the primary CD4+ T helper cell stimulating AE37 peptide vaccine may increase the number of activated CD8+ CTLs [26]. And Perez et al. demonstrated vaccination with AE37 primes not only the CD4+ T cells, but also primes CD8+ T cells and is able to induce CD8+ responses to both AE36 and AE37 in cancer patients [27]. AE37 is able to directly stimulate the HLA-DR alleles with epitopes present in the HER2 protein. The immunologic effect of AE37 vaccination has also been shown to increase IFN-γ + CD4 + responder cells which in turn assists in strong in vivo and in vitro autologous CTL lysing of tumor cells [28, 29]. Thus, the addition of the Ii-Key in AE37 specifically enhances immune responses via the MHC class I pathway [27].

The stimulation of both a CD4+ T helper cell and CD8+ CTL responses suggest that the AE37 peptide vaccine may also have synergistic effect in combination with other short peptide vaccines, which work primarily in a CD8+ CTL mediated fashion. A similar finding was demonstrated in a HER2 peptide derived vaccine on a dendritic cell platform that stimulates both CD8+ and CD4+ T cells. This MHC class 2 vaccine was also given in combination with anti-PD-1 therapy and demonstrated improved survival in a preclinical model [30]. It is worth exploring future trials of combinations of check point inhibitors and peptide-based vaccine strategies to improve DFS. While the potential for CTL-mediated, anti-tumor cytolytic effect via peptide vaccines like nelipepimut-S and GP2 certainly provide promise as a potential stand-alone weapon in the fight against cancer, the CTL effects are limited temporally given the natural transient course of such cytotoxic immune responses. Thus, a vaccine that combines CTL and T helper cell targeted peptides may not only induce the more immediate CTL-mediated cytolytic response against any occult residual disease, but also induce T helper cell-mediated long-term immunologic memory to protect against tumor recurrence.

## Conclusion

From checkpoint inhibitors to peptide vaccines, cancer immunotherapy is becoming ever more intricate as our understanding of subtypes of malignancies improves, and we better understand how we can help the body’s own defense system to fight active disease and prevent recurrences. Ultimately, our goal is to advance the field of personalized immunotherapies based on a patient’s specific disease characteristics. While neither vaccine demonstrated a statistically significant DFS benefit in the overall study population, there are signals of benefit in certain subpopulations of breast cancer patients. This is, perhaps, not surprising given distinct differences in terms of prognosis and treatment response in the different subtypes of breast cancer. This is reflected in our data, which suggests that different peptide vaccine strategies may be required to achieve clinical benefit for distinct subtypes of the same malignancy. Given our encouraging findings, additional randomized trials of the GP2 and AE37 peptide vaccines given independently for specific subsets as well as in combination warrant further investigation.

## Abbreviations

CG: Control group
CI: 95% Confidence interval
CTL: Cytotoxic T-lymphocyte
DFS: Disease-free survival
GM-CSF: Granulocyte–macrophage colony-stimulating factor
HER2 UE: HER2 Under-expression
HLA: Human leukocyte antigen
HR: Hazard ratio
ITT: Intention to treat
IQR: Interquartile range
MHC: Major histocompatibility complex
PT: Per treatment
TNBC: Triple-negative breast cancer
VG: Vaccine group

## References

[1] Aaltomaa S, Lipponen P, Eskelinen M, Kosma VM, Marin S, Alhava E, Syrjanen K (1992). Lymphocyte infiltrates as a prognostic variable in female breast cancer. Eur J Cancer.

[2] McArthur HL, Page DB (2016). Immunotherapy for the treatment of breast cancer: checkpoint blockade, cancer vaccines, and future directions in combination immunotherapy. Clin Adv Hematol Oncol.

[3] Fortis SP, Sofopoulos M, Sotiriadou NN, Haritos C, Vaxevanis CK, Anastasopoulou EA, Janssen N, Arnogiannaki N, Ardavanis A, Pawelec G, Perez SA, Baxevanis CN (2017). Differential intratumoral distributions of CD8 and CD163 immune cells as prognostic biomarkers in breast cancer. J Immunother Cancer.

[4] Slamon DJ, Godolphin W, Jones LA, Holt JA, Wong SG, Keith DE, Levin WJ, Stuart SG, Udove J, Ullrich A (1989). Studies of the HER-2/neu proto-oncogene in human breast and ovarian cancer. Science.

[5] Tovey SM, Reeves JR, Stanton P, Ozanne BW, Bartlett JM, Cooke TG (2006). Low expression of HER2 protein in breast cancer is biologically significant. J Pathol.

[6] Clynes RA, Towers TL, Presta LG, Ravetch JV (2000). Inhibitory Fc receptors modulate in vivo cytotoxicity against tumor targets. Nat Med.

[7] Mittendorf EA, Ardavanis A, Symanowski J, Murray JL, Shumway NM, Litton JK, Hale DF, Perez SA, Anastasopoulou EA, Pistamaltzian NF, Ponniah S, Baxevanis CN, von Hofe E, Papamichail M, Peoples GE (2016). Primary analysis of a prospective, randomized, single-blinded phase II trial evaluating the HER2 peptide AE37 vaccine in breast cancer patients to prevent recurrence. Ann Oncol.

[8] Peoples GE, Holmes JP, Hueman MT, Mittendorf EA, Amin A, Khoo S, Dehqanzada ZA, Gurney JM, Woll MM, Ryan GB, Storrer CE, Craig D, Ioannides CG, Ponniah S (2008). Combined clinical trial results of a HER2/neu (E75) vaccine for the prevention of recurrence in high-risk breast cancer patients: U.S. Military Cancer Institute Clinical Trials Group Study I-01 and I-02. Clin Cancer Res.

[9] Mittendorf EA, Clifton GT, Holmes JP, Schneble E, van Echo D, Ponniah S, Peoples GE (2014). Final report of the phase I/II clinical trial of the E75 (nelipepimut-S) vaccine with booster inoculations to prevent disease recurrence in high-risk breast cancer patients. Ann Oncol.

[10] Mittendorf EA, Lu B, Melisko M, Price Hiller J, Bondarenko I, Brunt AM, Sergii G, Petrakova K, Peoples GE (2019). Efficacy and safety analysis of Nelipepimut-S vaccine to prevent breast cancer recurrence: a randomized multicenter, phase III clinical trial. Clin Cancer Res.

[11] Knutson KL, Disis ML (2006). Augmenting T Helper Cell Immunity in Cancer. Curr Drug Targets.

[12] Knutson KL, Disis ML (2005). Tumor antigen-specific T helper cells in cancer immunity and immunotherapy. Cancer Immunol Immunother.

[13] Zanetti M (2015). Tapping CD4 T cells for cancer immunotherapy: the choice of personalized genomics. J Immunol.

[14] Schwartz RH (2003). T cell anergy. Annu Rev Immunol.

[15] Gillogly ME, Kallinteris NL, Xu M, Gulfo J, Humphreys RE, Murray J (2004). Ii-Key/HER-2/ neu MHC class-II antigenic epitope vaccine peptide for breast cancer. Cancer Immunol Immunother.

[16] Mittendorf EA, Storrer CE, Foley RJ, Harris K, Jama Y, Shriver CD, Ponniah S, Peoples GE (2006). Evaluation of the HER2/neu-derived peptide GP2 for use in a peptide-based breast cancer vaccine trial. Cancer.

[17] Mittendorf EA, Ardavanis A, Litton JK, Shumway NM, Hale DF, Murray JL, Perez SA, Ponniah S, Baxevanis CN, Papamichail M, Peoples GE (2016). Primary analysis of a prospective, randomized, single-blinded phase II trial evaluating the HER2 peptide GP2 vaccine in breast cancer patients to prevent recurrence. Oncotarget.

[18] Ann Mittendorf E, Perez S, Tzonis P, Pistamaltzian N, Anastasopoulou EM, Peace KJ, Vreeland T, Hale D, Clifton G, Litton J, Hofe E, Ardavanis A, Papamichail M, Earl Peoples G (2017). Subgroup efficacy evaluation of the AE37 HER2 vaccine in breast cancer patients in the adjuvant setting. J Clin Oncol.

[19] Jackson DO, Trappey FA, Clifton GT, Vreeland TJ, Peace KM, Hale DF, Litton JK, Murray JL, Perez SA, Papamichail M, Mittendorf EA, Peoples GE (2018). Effects of HLA status and HER2 status on outcomes in breast cancer patients at risk for recurrence—implications for vaccine trial design. Clin Immunol (Orlando, Fla).

[20] Pusztai L, Karn T, Safonov A, Abu-Khalaf MM, Bianchini G (2016). New Strategies in breast cancer: immunotherapy. Clin Cancer Res.

[21] Engels CC, Fontein DB, Kuppen PJ, de Kruijf EM, Smit VT, Nortier JW, Liefers GJ, van de Velde CJ, Bastiaannet E (2014). Immunological subtypes in breast cancer are prognostic for invasive ductal but not for invasive lobular breast carcinoma. Br J Cancer.

[22] Mittendorf EA, Storrer CE, Shriver CD, Ponniah S, Peoples GE (2006). Investigating the combination of trastuzumab and HER2/neu peptide vaccines for the treatment of breast cancer. Ann Surg Oncol.

[23] Hickerson AM, Clifton GT, Hale DF, Peace KM, Holmes JP, Vreeland TJ, Litton JK, Murthy RK, Lukas JJ, Mittendorf EA (2019) Peoples GE Final analysis of Nelipepimut-S plus GM-CSF with trastuzumab versus trastuzumab alone to prevent recurrences in high-risk, HER2 low-expressing breast cancer: A prospective, randomized, blinded, multicenter phase IIb trial. In: ASCO-SITC Clinical Immuno-Oncology Symposium, San Francisco, CA, March 2, 2019

[24] Disis ML, Grabstein KH, Sleath PR, Cheever MA (1999). Generation of immunity to the HER-2/neu oncogenic protein in patients with breast and ovarian cancer using a peptide-based vaccine. Clin Cancer Res.

[25] Holmes JP, Benavides LC, Gates JD, Carmichael MG, Hueman MT, Mittendorf EA, Murray JL, Amin A, Craig D, von Hofe E, Ponniah S, Peoples GE (2008). Results of the first phase I clinical trial of the novel II-key hybrid preventive HER-2/neu peptide (AE37) vaccine. J Clin.

[26] Gates JD, Clifton GT, Benavides LC, Sears AK, Carmichael MG, Hueman MT, Holmes JP, Jama YH, Mursal M, Zacharia A, Ciano K, Khoo S, Stojadinovic A, Ponniah S, Peoples GE (2010). Circulating regulatory T cells (CD4+CD25+FOXP3+) decrease in breast cancer patients after vaccination with a modified MHC class II HER2/neu (AE37) peptide. Vaccine.

[27] Perez SA, Kallinteris NL, Bisias S, Tzonis PK, Georgakopoulou K, Varla-Leftherioti M, Papamichail M, Thanos A, von Hofe E, Baxevanis CN (2010). Results from a phase I clinical study of the novel Ii-Key/HER-2/neu(776–790) hybrid peptide vaccine in patients with prostate cancer. Clin Cancer Res.

[28] Sotiriadou NN, Kallinteris NL, Gritzapis AD, Voutsas IF, Papamichail M, von Hofe E, Humphreys RE, Pavlis T, Perez SA, Baxevanis CN (2007). Ii-Key/HER-2/neu(776–790) hybrid peptides induce more effective immunological responses over the native peptide in lymphocyte cultures from patients with HER-2/neu+ tumors. Cancer Immunol Immunother.

[29] Voutsas IF, Gritzapis AD, Mahaira LG, Salagianni M, von Hofe E, Kallinteris NL, Baxevanis CN (2007). Induction of potent CD4+ T cell-mediated antitumor responses by a helper HER-2/neu peptide linked to the Ii-Key moiety of the invariant chain. Int J Cancer.

[30] Kodumudi KN, Ramamoorthi G, Snyder C, Basu A, Jia Y, Awshah S, Beyer AP, Wiener D, Lam L, Zhang H, Greene MI, Costa RLB, Czerniecki BJ (2019). Sequential Anti-PD1 therapy following dendritic cell vaccination improves survival in a HER2 mammary carcinoma model and identifies a critical role for CD4 T cells in mediating the response. Front Immunol.

